# A global analysis of the inclusion of wildlife in national action plans for antimicrobial resistance

**DOI:** 10.1093/jacamr/dlag100

**Published:** 2026-06-08

**Authors:** Nicholas Bor, Jessica Mitchell

**Affiliations:** Division of Global Agriculture and Food Systems, Royal (Dick) School of Veterinary Studies, University of Edinburgh, Edinburgh, UK; Division of Global Agriculture and Food Systems, Royal (Dick) School of Veterinary Studies, University of Edinburgh, Edinburgh, UK

## Abstract

**Background:**

Antimicrobial resistance (AMR) is a growing global health threat to humans, domestic animals and wildlife. In response, the WHO endorsed the AMR Global Action Plan in 2015 to guide the development of national action plans (NAPs). NAPs articulate priorities, sectoral roles and implementation strategies to address AMR. Although the wildlife sector has been noted to be minimally included in these documents, the extent and nature of its inclusion remain unclear. This is concerning given wildlife’s role at the human–animal–environment interface in AMR dynamics.

**Objectives:**

To assess the extent and context of wildlife inclusion in AMR NAPs.

**Methods:**

We reviewed 177 AMR NAPs available in the WHO library. Each document was searched for the terms ‘wildlife’ or ‘wild’, and the extracted excerpts were analysed using an inductive thematic analysis to determine the context in which wildlife was referenced.

**Results:**

Wildlife was minimally included in the analysed NAPs, with only 11% (20/177) containing wildlife-related references. Fifty-eight mentions were identified across documents, appearing within One Health definitions, proposed actions, document headings and governance structures. Surveillance was the most proposed wildlife-related activity; however, detailed implementation strategies were absent. Uganda was the only country whose NAP explicitly incorporated a wildlife-associated institution within its governance framework.

**Conclusions:**

Wildlife is minimally included in AMR NAPs, with references predominantly conceptual rather than operational. The absence of defined implementation mechanisms undermines effective One Health integration and risks weakening comprehensive AMR responses.

## Introduction

Antimicrobial resistance (AMR) represents one of the most severe and escalating global health threats of the 21st century. AMR refers to the ability of microorganisms to withstand the effects of antimicrobial agents designed to kill or inhibit their growth. It is a slow, natural evolutionary process that predates the use of modern antimicrobials.^1,2^ This process has been accelerated by anthropogenic activities such as the misuse and overuse of antimicrobials—practices that are often exacerbated by high infectious disease burdens. Broader societal and structural conditions such as poverty, inadequate sanitation and weak healthcare systems compound these pressures, fostering environments where resistance may emerge and spread more readily.^3^ Resistance spreads across human, animal and environmental sectors. The One Health approach recognizes the interconnectedness of these three health domains and has been widely acknowledged as a holistic approach to addressing AMR.^2^

In response to the threat of AMR, the WHO instigated the development of the AMR global action plan (GAP).^4^ This plan was endorsed in May 2015 during the 68th World Health Assembly and contained five strategic objectives for addressing AMR. These include the following: (i) improving awareness and understanding of AMR; (ii) strengthening knowledge through surveillance and research; (iii) reducing the incidence of infection through effective sanitation, hygiene and infection prevention and control (IPC); (iv) optimizing the use of antimicrobials in health sectors; and (v) developing the economic case for sustainable investment in AMR interventions.^4^ The GAP has been instrumental in supporting WHO-affiliated countries to build their own AMR national action plans (NAPs), which are country-level frameworks that align with AMR GAP’s strategic objectives.^5^

Previous assessments of AMR NAPs have identified specific gaps in sectoral representation; Caputo *et al*. (2022) found that 37% of NAPs failed to mention aquaculture,^6^ while Charani *et al*. (2023) reported that most NAPs focused on developing new antimicrobials for human use, rather than strengthening stewardship to preserve the efficacy of existing antimicrobials across One Health.^6,7^ A recent regional analysis of NAPs from Europe and Latin America has also revealed minimal inclusion of wildlife. Just 6 out of 44 European NAPs and 1 out of 22 NAPs from the Latin American region included discussion of wildlife.^8^

The findings from these two regions reinforce the broader evidence that wildlife remains largely excluded from AMR policy frameworks, which tend to prioritize humans, livestock, crops and their immediate environments. Wildlife is only considered when in contact with humans.^9^ This is concerning given the increasing reports of AMR in free-ranging wildlife.^10^ Long-distance wildlife migrants have been described as potential disseminators of resistance genes.^11^ Critically important resistant genes such as extended-spectrum beta-lactamase (ESBL)–producing and carbapenem-resistant (CARBA) Enterobacterales have been isolated in wildlife populations. These pose potential risks to human and animal health, including the transmission of resistant genes across species and the reduced effectiveness of critically important antimicrobial agents.^8^

The omission of wildlife undermines the very foundation of the One Health approach. Wildlife occupies a unique ecological interface, inhabiting both natural and human-dominated environments, and intersecting human, animal and environmental health systems.^12,13^ Highlighting the role of wildlife in AMR dynamics is not intended to vilify them, as resistance has been documented across all One Health sectors.^14^ Singling out wildlife as disseminators of resistance risks misdirecting attention, yet what is needed is a balanced and inclusive multisectoral collaboration.

While regional analysis suggests minimal inclusion of wildlife, no global analysis has systematically evaluated the inclusion of wildlife in AMR NAPs. This study aims to fill this gap by evaluating the inclusion of wildlife in AMR NAPs globally, quantifying the extent of its inclusion and characterizing the contexts in which wildlife is mentioned.

## Methods

### Data source and search strategy

AMR NAPs available in the WHO library^4^ (as of November 2025) were screened for wildlife-related terminology using the document search function. Prior to full screening, we reviewed a subset of NAPs to identify how wildlife was described and to refine the search terms. As NAPs did not consistently define or categorize wildlife using standardized terminology (e.g. farmed, free-ranging or captive), we selected broad search terms to maximize sensitivity in capturing relevant references. The key search terms used were ‘wildlife’ and ‘wild’. If any of these terms were present in the NAP, the full sentence containing the term was extracted and copied into a Microsoft Word document for subsequent thematic analysis. NAPs written in languages other than English were translated into English using Google Cloud Translation. The same search and extraction process was then applied to the translated NAPs. To quantify the extent of wildlife inclusion, the number of NAPs containing the search terms was recorded. To ensure that relevant terms were not missed due to translation issues, native speakers within the department verified whether these terms were present in the original NAPs.

### Analysis of the wildlife mentions

An inductive thematic analysis was conducted using Braun and Clarke’s framework.^15^ This involved repeated reading of the extracted wildlife quotes to achieve data familiarization, while returning to the sections of the NAPs where the quotes were extracted to determine context. Codes were generated inductively by assigning descriptive labels that reflected their content and context. Codes were then grouped to identify broader patterns across the dataset, leading to the development of candidate themes. These themes were subsequently reviewed and refined to ensure coherence and clear distinctions between themes. Final themes were defined and named to accurately reflect their scope and meaning.

Data extraction, coding and initial theme development were conducted by a single reviewer (NB). To minimize potential bias associated with single-coder analysis, theme review, refinement and validation were conducted collaboratively by two reviewers (N.B. and J.M.).

All data extraction, coding and thematic development were conducted manually using Microsoft Word. No specialized qualitative data analysis software was used due to the relatively small and manageable dataset.

## Results

### Descriptive analysis

**Figure 1.**
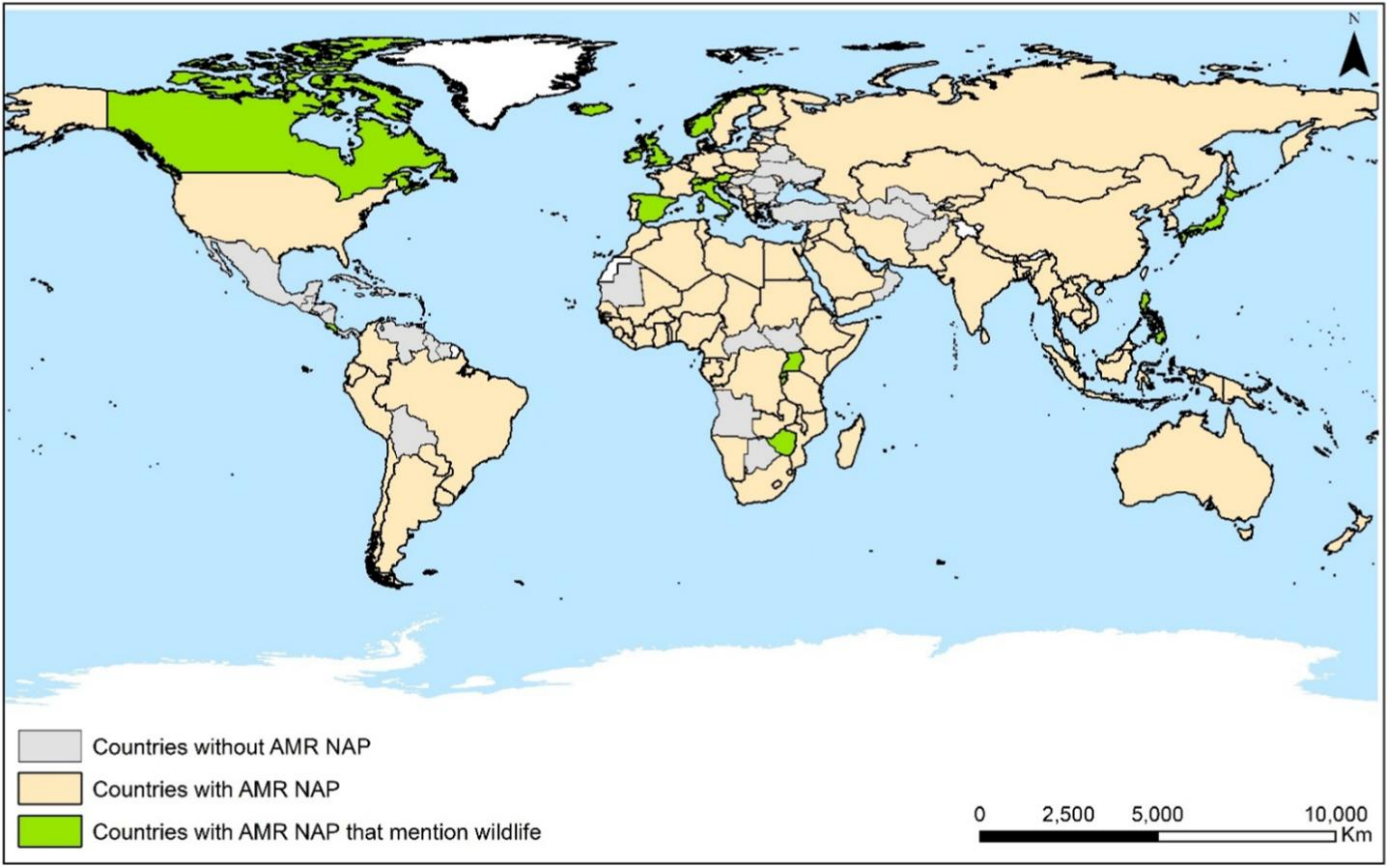
Global distribution of AMR NAPs and inclusion of wildlife.

A total of 177 NAPs from 145 countries were reviewed (Figure 1). These represented all six WHO-defined global regions, namely, Africa, America, Eastern Mediterranean, Europe, South-East Asia and Western Pacific.^4^ Wildlife was mentioned 58 times within 20/177 (11%) NAPs (Table 1). None of the countries from the Eastern Mediterranean and South-East Asia regions mentioned wildlife in their NAPs. Of the 20 NAPs that mentioned wildlife, 7 (35%) were up-to-date with their implementation periods ending in 2025 or later and 15 (75%) were due for review in 2026 (Table 2).

**Table 1.** Summary of the reviewed AMR NAPs with wildlife mentions

WHO Regions^4^	Number of Countries in WHO Regions	Number of NAPs Reviewed	NAPs with Wildlife Mentions	Number of Wildlife Mentions
Africa	37	44	5	32
Americas	14	16	3	4
Eastern Mediterranean	21	22	0	0
Europe	38	48	8	16
South-East Asia	11	13	0	0
Western Pacific	24	34	4	6
Total	145	177	20	58

**Table 2.** Specific country NAPs and number of wildlife mentions

Region	Country	Implementation Years	No of Mentions
Africa	Burundi	2020–23	6
	Rwanda	2020–24	2
	Uganda	2018–23	8
	Uganda	2024–29	14
	Zimbabwe	2017–21	2
America	Canada	2017	1
	Canada	2023–28	1
	Costa Rica	2018–22	2
Europe	Austria	2021	1
	Iceland	2022	1
	Ireland	2021–25	1
	Italy	2022–25	5
	Norway	2015–20	1
	Spain	2019–21	1
	Switzerland	2015	2
	UK	2024–29	4
Western Pacific	Japan	2023–27	2
	Philippines	2019–23	1
	Philippines	2024–28	2
	Singapore	2018–20	1
Total			58

### Thematic analysis of wildlife mentions

Themes that emerged from the analysis of the wildlife mentions were definitions, proposed actions, subtitles and governance (Table 3).

**Table 3.** Themes and subthemes of the wildlife mentions

Themes	Subthemes	Count	Percentage Count
Definitions	One Health, Zoonosis, AMR	23	40%
Proposed actions	Surveillance, Awareness, AMU	18	31%
Governance	AMR committee	10	17%
Subtitles	Surveillance, Awareness, AMU	7	12%
Total		58	100

### Definition theme

There were 23 instances where wildlife was referenced in the context of One Health, zoonotic and AMR definitions across 10 NAPs. These definitions highlighted the interconnectedness of the environment, human and animal sectors. However, none of the reviewed NAPs provided an explicit definition of wildlife. All the One Health definitions were from the UK, Italy, Switzerland and Spain and appeared primarily in the introduction sections of the NAPs:

‘One Health is an integrated, unifying approach that aims to sustainably balance and optimise the health of humans, animals, plants and ecosystems. It recognises the close links between the health of humans, domestic and wild animals, plants and the wider environment (including ecosystems). Drug-resistant microbes can be found in people, animals, food and in the environment. Through contact with other people, animals and the environment, these microbes and resistance mechanisms can be transferred between and among different species, affecting the health of people and animals, including companion animals, food-producing animals, and wildlife,’ United Kingdom 2024–2028.

Zoonotic definitions from Italy and the Philippines mentioned wildlife in the context of stating that many zoonotic pathogens have a wildlife origin:

‘Over 70% of human infectious diseases known today have a zoonotic origin, that is, they are transmitted by animals and 60% of these have been transmitted by wild animals,’ Italy 2022–2025.

AMR definitions mentioned wildlife with regard to how resistance develops and spreads across the One Health domains including wildlife:

‘This process, known as horizontal gene transfer, occurs when an exchange of genetic material among bacteria takes place, i.e. on and among people, wild animals, livestock, pets or in the environment,’ Switzerland 2015.

### Proposed action themes

There were 18 wildlife mentions related to proposed actions for tackling AMR across 10 NAPs. The proposals included conducting surveillance, evaluating awareness among wildlife stakeholders and developing antimicrobial use (AMU) guidelines for the wildlife sector. These three subthemes are discussed below.

#### Surveillance subtheme

Surveillance was the most mentioned subtheme within the proposed action theme. These NAPs were from Burundi, Rwanda, Uganda, the Philippines, Singapore and Zimbabwe. In this context, surveillance is understood as systematic collection, analysis and reporting of AMR data across human, animal and environmental sectors. On the other hand, monitoring refers to periodic tracking of specific indicators.^16^ Across the reviewed NAPs, there were calls to develop standard operating procedures for surveillance activities for clinical and post-mortem samples from both domestic and wild animals:

‘Analyse, disseminate and share surveillance data and information to facilitate decision making on diagnoses and treatment in clinical public health, veterinary practices, environment and wildlife laboratories and food technologies,’ Rwanda 2020–2024.

Some NAPs also acknowledged gaps in AMR surveillance, especially in environmental and wildlife domains. Canada’s 2017 NAP noted limited surveillance of resistance in the environmental pathways such as soil, water and wildlife. Similarly, Ireland’s NAP proposed an increased understanding of the role of wildlife in transmitting resistance:

‘The role of native Irish wildlife species in transference is not well understood,’ Ireland 2021–2025.

#### Knowledge and awareness subtheme

In terms of awareness and knowledge, Zimbabwe’s NAP of 2017–21 proposed conducting knowledge, attitudes and practices (KAP) survey on infection, prevention and control among veterinary personnel, including wildlife veterinarians from government and private sectors:

‘Conduct a KAP survey on IPC, good animal health management practices, and biosecurity among veterinary personnel in private and government sectors/environmental officers/food safety officials/wildlife veterinarians,’ Zimbabwe 2017–2021.

#### Antimicrobial use subtheme

The Costa Rica NAP called for the drafting of AMU guidelines for agriculture and wildlife facilities whilst Iceland’s NAP already monitors antimicrobial use in all species:

‘Draft generic mandates on the use of antimicrobials in primary production (agricultural) farms and urban wildlife care facilities,’ Costa Rica 2018–2022.

### Subtitle theme

Seven wildlife mentions were found within the subtitle sections of three NAPs from Burundi, Uganda and Rwanda in the African region. These subtitles preceded text on the proposed actions.

### Governance theme

Ten mentions of wildlife pertained to governance. All were from the Uganda NAP where Uganda Wildlife Authority^17^ is a signatory. Uganda was the only country to have a wildlife-affiliated organization as a member of the national AMR Sub-Committee responsible for overseeing NAP implementation:

‘In line with the recognition of the importance of a One Health Approach, the National Action Plan shall be coordinated by the National One Health Platform (NOHP). The NOHP is a collaboration between the Ministry of Health, Ministry of Agriculture Animal Health and Fisheries, the Ministry of Water and Environment and the Uganda Wildlife Authority through a Memorandum of Understanding with the objective of coordinating joint efforts to address health issues that affect all the sectors,’ Uganda 2024–2029.

## Discussion

This study demonstrates the limited inclusion of the wildlife sector in AMR NAPs globally. Only 11% of the reviewed NAPs contained any mention of wildlife. These mentions were mostly framed at a conceptual level rather than as actionable strategies. Wildlife was primarily mentioned within One Health definitions and proposed actions, with limited attention to implementation mechanisms. Surveillance was frequently cited as a proposed action; however, in the NAPs that included wildlife, it was rarely accompanied by explicit implementation or operational details, with some plans acknowledging it as an existing gap.

The limited inclusion of wildlife observed in this study reflects a long-standing imbalance in sectoral representation within AMR policy frameworks. AMR NAPs are intended to adopt a One Health approach that integrates human, animal and environmental sectors.^18^ Iossa and White reported that the environmental sector was largely missing from AMR NAPs, with most plans focusing on human health and livestock production systems.^19^ Such sectoral imbalance is further reflected in weak multisectoral collaboration, with only 17% of NAPs demonstrating strong integration across human, veterinary and agricultural sectors.^5^

This underrepresentation of the environmental sector extends to wildlife within a One Health framework. The environment encompasses living and non-living components that interact with natural systems, including climate, ecosystems, and biological communities.^20^ Wildlife, as part of these ecological systems, can therefore be understood as an integral component of the environmental domain. From this perspective, the limited integration of wildlife in AMR NAPs reflects a broader pattern of environmental exclusion rather than an isolated oversight, highlighting a systemic gap in AMR governance. This interpretation aligns with regional analyses reporting minimal inclusion of wildlife in AMR policy frameworks,^8^ reinforcing the view that wildlife exclusion reflects a broader pattern of environmental underrepresentation.

Although surveillance was the most frequently mentioned subtheme, it was largely presented as a proposal without an actionable strategy and was sometimes conflated with monitoring. This contrasts sharply with human health AMR surveillance, which has standardized reporting mechanisms.^21^ For example, the Global Antimicrobial Resistance and Use Surveillance System (GLASS) aggregates AMR and AMU data from clinical settings to inform action.^22^ The limited wildlife AMR surveillance may be due to historical prioritization of clinical and agricultural settings, where risks to human health and economic productivity have been the primary focus.^9,23^ Additional barriers to the utilization of wildlife in AMR surveillance include logistical difficulties in sampling migratory wild species, limited funding and competing public health priorities. These challenges are particularly pronounced in resource-limited settings, where surveillance capacity is constrained by scarce financial resources.^24^ Furthermore, there is a need for standardized, multidisciplinary approaches to optimize wildlife AMR surveillance, including non-invasive sampling, integration of databases and advanced analytical techniques to understand transmission pathways across humans, livestock and wildlife sectors.^25^

The absence of actionable wildlife surveillance is problematic given the ecological role of wildlife in AMR dynamics. Wildlife occupies a critical interface between human, animal and environmental sectors,^12^ where they may be exposed to resistant determinants circulating across sectors and may act either as reservoirs or disseminators or sentinels of resistant genes.^23^ When these resistant genes enter the environment, they can interact with other resistance determinants, promoting the spread of resistance across microbial populations.^26^ Excluding wildlife from surveillance systems therefore limits the ability to capture these cross-sectoral transmission dynamics, resulting in an incomplete understanding of AMR within a One Health framework.

All governance-related mentions of wildlife in this analysis were found in Uganda's NAPs where Uganda Wildlife Authority^17^ was a key signatory in the national plans. UWA is a significant government-supported institution that was militarized to protect wildlife from poaching and illegal hunting.^27^ This inclusion may partly explain its visibility within Uganda’s key policy documents. Despite this formal inclusion, Uganda’s NAP lacked clearly defined and actionable wildlife-related AMR activities. This suggests that formal inclusion of wildlife institutions in AMR frameworks does not automatically translate into operational implementation. Previous studies note that low-income countries often replicate global strategic objectives with limited implementation, whereas high-income countries develop context-specific priorities with stronger implementation.^28^ In our analysis, Australia’s NAP made no formal reference to wildlife. However, this country has recently developed antimicrobial stewardship guidance specific to wildlife. This document contains guidelines on appropriate antimicrobial use to curb practices that may contribute to the development of resistance.^29^ These observations show that effective AMR governance requires both formal inclusion of wildlife institutions in the NAPs and detailed operational strategies.

The limited inclusion of wildlife in AMR NAPs is partly attributable to limited AMR research in wildlife. A review of 158 mpa##8202;616 AMR studies found that most of these studies focused on clinical and public health outcomes for human beings. The key themes discussed were antibiotic stewardship, multidrug-resistant tuberculosis and novel approaches like nanoparticle-based therapies.^30^ Similarly, a bibliometric analysis of AMR studies for a 40 year period found only 219 wildlife-related studies. These studies lacked global representation as most publications originated from Spain, Portugal, the USA and the UK.^10^ Furthermore, these studies have common limitations such as small sample sizes, inconsistent resistance detection methods, fragmented surveillance data, regional bias in wildlife AMR research, reliance on point-prevalence and convenience sampling.^10,11,31,32^ Collectively, these evidence gaps may constrain countries’ ability to understand the magnitude of the problem for better prioritization and inclusion. Strengthening the research base on antimicrobial use and resistance in wildlife is therefore critical to informing more robust evidence-based policy integration.

A key limitation of this paper is the temporal validity of the analysed NAPs. At the time of analysis, 75% had either surpassed or were nearing the end of their implementation periods and were therefore due for revision. In addition, the NAPs were developed across differing timeframes with varying implementation periods. This limits direct comparability between countries as priorities and guidance may have evolved over time. Theoretically, the gaps identified in this analysis could be addressed in the next iterations. However, meaningful incorporation of wildlife will only be achieved if discussions and actions begin imminently. Similarly, the recent call by Quadripartite organizations to revise the AMR GAP may have presented an opportunity to strengthen the integration of wildlife in global and national AMR frameworks.^33^ Although this call has now closed, its outcomes are yet to be seen when the plans have been rolled out. Previous reviews of AMR NAPs indicate that the strategic objectives in the national plans closely aligned with those in the global plan,^5^ suggesting that revision at the global level may have cascading effects on how wildlife is addressed and incorporated at the national level. Another limitation was the absence of an explicit definition of wildlife in the reviewed NAPs. This makes it difficult to determine whether the key search terms may have missed relevant references, as wildlife may be conceptualized or contextualized differently across nations or regions. It is possible that some may use alternative terms such as non-domesticated or free-ranging animals to refer to wildlife although our pre-screening phase did not find any evidence of this alternative terminology.

Building on the gaps identified in this analysis, we recommend that future NAPs should move beyond nominal mention of wildlife and inclusion of wildlife-associated institutions. Clear roles for wildlife-sector stakeholders, measurable objectives and coordinated surveillance and reporting mechanisms are essential to ensure that wildlife is meaningfully integrated into AMR governance and the One Health framework.

### Conclusion

This study highlights the limited and inconsistent integration of the wildlife sector within AMR NAPs. Only 11% of the reviewed NAPs mentioned wildlife, and where it was included, this was largely conceptual rather than operational. Wildlife was primarily referenced within One Health definitions, with limited attention to implementation mechanisms. Surveillance was commonly proposed without measurable or actionable wildlife-specific strategies. These findings are consistent with earlier assessments demonstrating weak multisectoral collaboration in AMR governance, despite broad alignment with global strategic objectives.

The analysis further demonstrates that formal recognition of wildlife within AMR frameworks does not necessarily translate into actionable outcomes. While Uganda’s NAP includes wildlife through institutional representation, this did not result in clearly defined AMR-related activities. On the other hand, Australia has developed wildlife-specific antimicrobial stewardship guidance despite the absence of wildlife in its NAP. These contrasts underscore a persistent gap between strategy and policy implementation. Given the documented presence of clinically relevant resistance genes in wildlife and the interconnected nature of AMR across One Health domains, addressing this gap will require moving beyond symbolic inclusion toward clearly defined roles, measurable objectives and actionable strategies. As global and national AMR strategies undergo revision, there is a timely opportunity to strengthen the meaningful integration of wildlife within AMR governance frameworks.
